# 3-D reconstruction of a human fetus with combined holoprosencephaly and cyclopia

**DOI:** 10.1186/1746-160X-5-14

**Published:** 2009-06-29

**Authors:** Wolfgang H Arnold, Veronika Meiselbach

**Affiliations:** 1 Department of Anatomy, Faculty of Dental Medicine, University of Witten/Herdecke, Alfred Herrhausenstrasse 50, 58448 Witten, Germany

## Abstract

**Background:**

The purpose of this study was to examine a human fetus with combined holoprosencephaly and cyclopia by means of histology and 3-D reconstruction to determine the internal structure and extent of the malformation.

**Methods:**

The head from a human fetus at 20 weeks gestation and a diagnosis of holoprosencephaly and cyclopia was investigated histologically and three-dimensionally reconstructed with CAD techniques. The cranial bones, blood vessels, nerves, eye and brain anlagen were reconstructed.

**Results:**

The 3-D reconstruction revealed both severe malformation and absence of the facial midline bones above the maxilla, and a malformation of the maxilla and sphenoid bone. The mandible, posterior cranial bones, cranial nerves and blood vessels were normal. A synophthalmic eye with two lenses was found. The prosencephalon was a single small protrusion above the diencephalon. No nasal cavity was present. Above the single eye a proboscis was found.

**Conclusion:**

The absence of the facial midline bones above the maxilla and the presence of a proboscis as a nose-like structure above the cyclopic eye both mean that there was a developmental defect in the fronto-nasal facial process of this fetus.

## Background

Holoprosencephaly is a developmental disturbance which is characterized by incomplete cleavage of the prosencephalon into two hemispheres and can also affect the midline structures of the face. The clinical expression of holoprosencephaly is extremely variable and suggests a complex interaction of developmental, genetic and environmental factors. The prevalence of holoprosencephaly is 1/16,000 live births with an incidence of 1/250 in first-trimester embryos [1]. The range of expression of holoprosencephaly varies from mild forms, wherein the right and left ventricles are separated, but there is continuity across the frontal cortex, to severe forms, where there is a single brain and no interhemispheric fissure (alobar holoprosencephaly). The severe forms are generally associated with facial deformities such as anophthalmia, cyclopia, and the presence of a proboscis. Clinical reports about newborn cases with mild forms of holoproscencephaly are abundant, whereas embryological documentation of the severe forms is scarce [2-9]. The most severe forms of holoprosencephaly are usually incompatible with postnatal life. For a better understanding of this malformation it is important to gain more embryological data.

The pathogenetic mechanisms of holoprosencephaly are still unknown, but both drug and alcohol abuse during early pregnancy as well as genetic defects have been described [1,7,10-13]. A number of genes have been discovered to be involved, including sonic hedgehog (SHH) [14,15], ZIC2 [16], SIX3 [17], TGIF [18], and others [1], and they are now considered to be important in this developmental defect. It is now assumed that multiple genetic hits and/or environmental exposures are required for the expression of holoprosencephaly [19,20]. Therefore, the multifactorial etiology of holoprosencephaly is considered to be the cause of the heterogeneity of the clinical severity of the malformation.

One characteristic feature of the facial deformities in holoprosencephaly is the defective structure in the median plane. It comprises one orbit with a single cyclopic eye (synophthalmia bilentica), a missing ethmoid complex, a proboscis above the eye, severe hypotelorism, midfacial hypoplasia, a midline cleft lip, the absence of nasal bones and a single upper incisor [14]. In the literature, the proboscis has been referred to as a nose-like structure [5,6,14,21], but so far no detailed investigation of this structure has been performed.

Human embryonic malformations may sometimes be regarded as normal variants, which allows for the explanation of some stages in normal development. In this respect, detailed investigations of developmental disturbances not only enlighten pathological conditions, but also give us hints about normal development.

The aim of this study was to investigate and describe in more detail the defective bone structures, nerves and blood supply of the facial and neural cranium of this fetus.

## Methods

Craniofacial tissues were studied in a spontaneously aborted human fetus of undetermined sex and an estimated age of 20 – 22 weeks. The maternal history was not available. Due to formalin fixation prior to receipt of the fetus, karyotyping was not possible.

After fixation in 10% formalin, the head was embedded in paraffin and serial histological sections in the horizontal plane were cut at a thickness of 10 μm. Every 10th section was collected and alternately stained with either hematoxylin – eosin or azan. Photomicrographs of every section were taken with a Nikon Coolpix 8400 camera with a resolution of 8 megapixels. In addition, the sections were studied with a Leitz DMRB microscope (Leica, Wetzlar, Germany) and additional microphotographs were taken. A computer program for 3-D reconstruction and rendering, AutoCAD 2009 (Autodesk Inc., USA), was used. The photographs of the sections were consecutively imported into AutoCAD 2009 and superimposed according to the method of the best fit. The outlines of the relevant structures were then digitized, each in separate layers. A total of 470 sections were digitized. Digitizing a single section took between 5 and 30 minutes depending on the complexity of the traced structures. From each structure, a 3-D meshwork wire frame image was created. By freezing or thawing single structures (electronic dissection), the three-dimensional relation of different bones, nerves, and blood vessels could be demonstrated.

## Results

### Gross anatomical features

**Figure 1:**
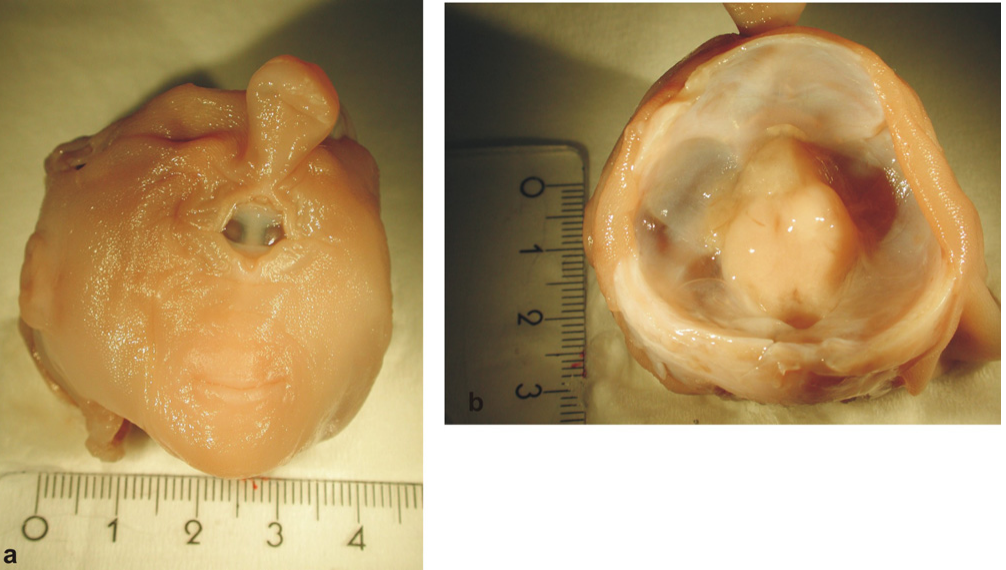
**Photographs of the macroscopic appearance of the head**. a) Frontal view of the investigated head. b) View of the opened cranium with remnants of brain.

The height of the head measured 5 cm, and its biparietal width was 4 cm. The cyclopic face showed synophthalmia with a single orbit (one eye with two pupils), an absent nose and a proboscis above the eye (Fig. 1a). The mouth was closed with no apparent clefting of the lips. After opening the cranium, the remnants of the brain were found, which appeared to be the diencephalon and a single prosencephalic prominence anterior to the diencephalon (Fig. 1b).

### Cranial bones

**Figure 2:**
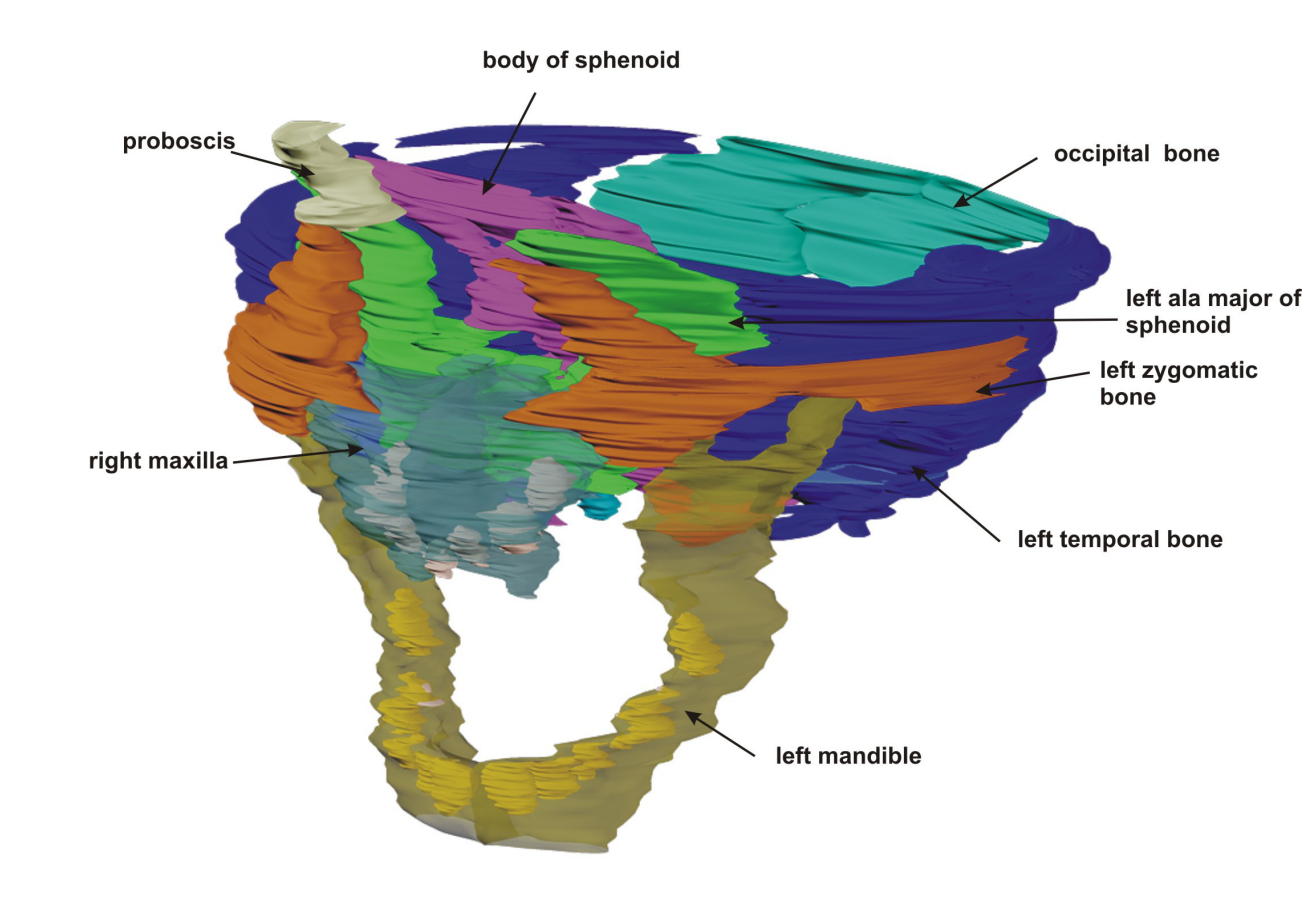
**Overview of cranial bones**. Rendered 3-D reconstruction of cranial bones, left oblique view. Mandible and maxilla are in transparent colors to show the tooth germs within the bones.

**Figure 3:**
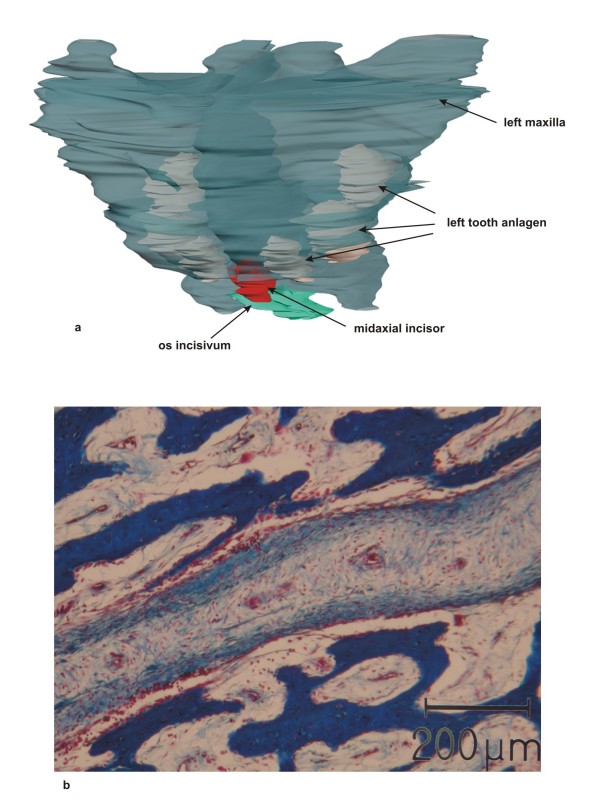
**Overview and histology of maxillary bone**. a) Rendered 3-D reconstruction of maxillary bones in transparent color depicting 3 tooth germs on each side and a single midaxial incisor. b) Histological section of the intermaxillary lamina with undifferentiated mesenchyme between the maxillary bones. Azan staining.

**Figure 4:**
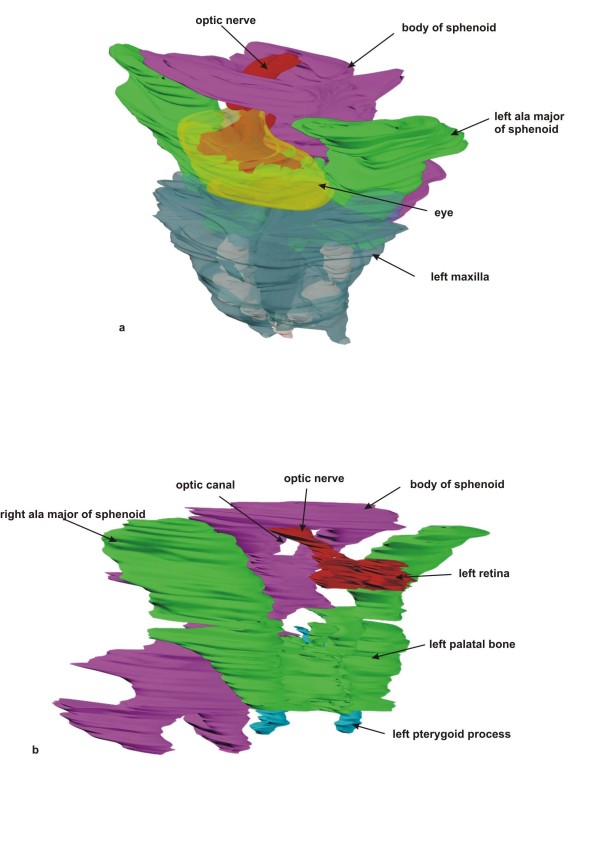
**Maxilla and sphenoid complex**. a) Rendered 3-D reconstruction of the maxillary complex and eye, left upper view. There is a single eye within the orbit containing two retinae. A single optic nerve leaves the orbit through a central optic canal. b) Rendered 3-D reconstruction of the sphenoid complex with the pterygoid, palatal bones, and sphenoid bone in the right view. The paired retinae and the optic nerve are also shown.

**Figure 5:**
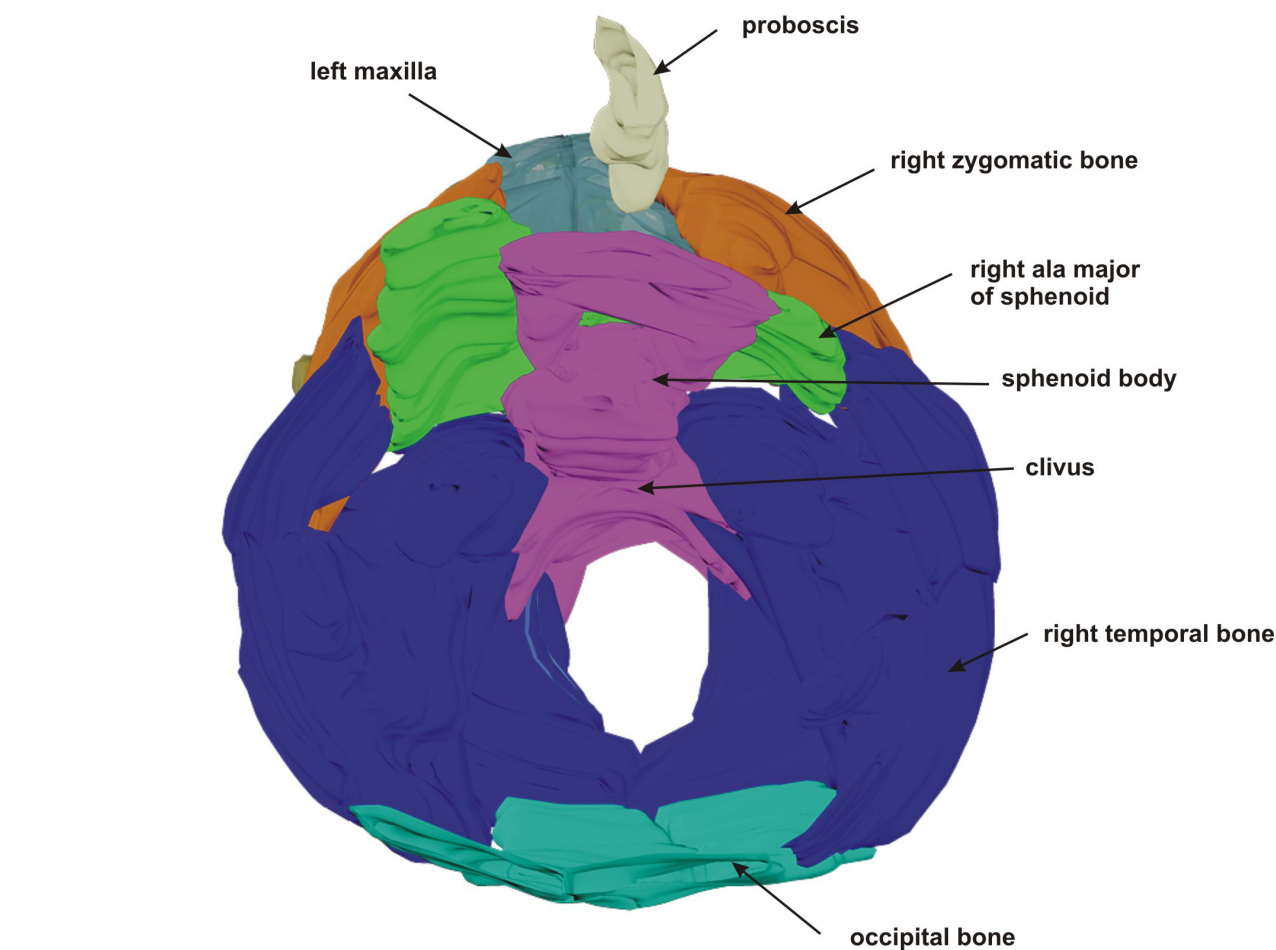
**Overview of cranial base**. Rendered 3-D reconstruction of the inner cranial base, upper view. In the frontal part, the ethmoid is missing and there is a single optic canal in the body of the sphenoid.

The mandible appeared to be normally developed, with five tooth anlagen on each side (Fig. 2). Both maxillae were hypoplastic, with three tooth anlagen on each side. Between the maxillae the anlage of a single midaxial incisor was found (Fig. 3a). No nasal septum or nasal cavity was present; instead, between both maxillae, a small lamina of undifferentiated mesenchyme was demonstrated histologically (Fig. 3b). Palatal bones, the sphenoid body and the alae majores of the sphenoid appeared to be normally developed. However, only one optic canal with a single optic nerve was found anterior to the sella turcica. The alae minores of the sphenoid were absent (Fig. 4). No ethmoid bone could be detected. The clivus zygomatic and the temporal bones, including the middle and posterior cranial cavity, showed no disturbances (Fig. 5). For comparison to normal anatomy, refer to Arnold et al. [3].

### Cranial nerves

**Figure 6:**
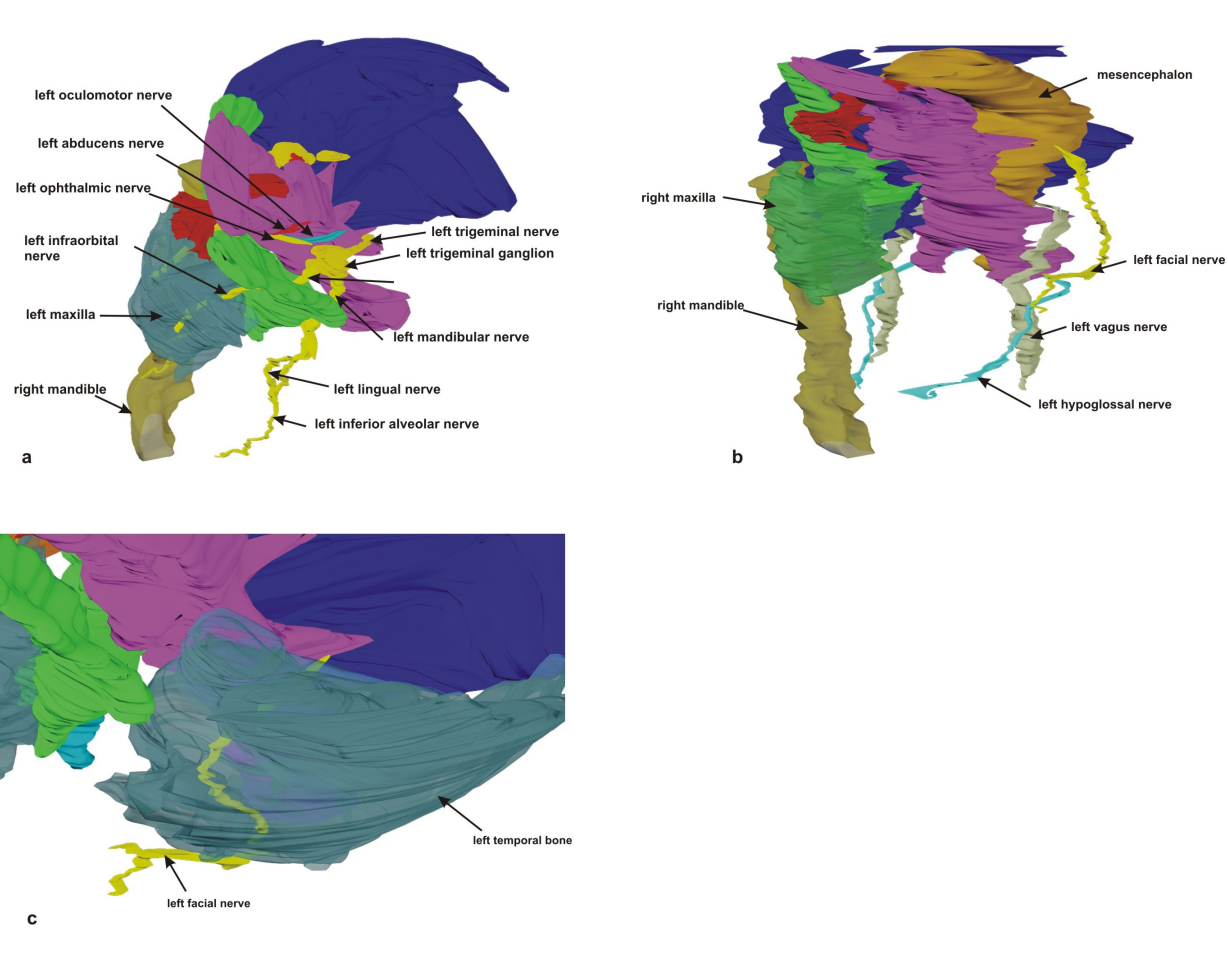
**Overview of the course of main cranial nerves**. a) Left oblique slight upper view of a rendered 3-D reconstruction of the abducens, oculomotor and trigeminal nerves. The left temporal bone and the left mandible have been removed. b) Left frontal view of a rendered 3-D reconstruction of the facial, vagus, and hypoglossal nerves. c) Left oblique upper view of the course of the facial nerve within the temporal bone.

From the cranial nerves the optic, oculomotor, abducens, trigeminal, facial, vagus and hypoglossal nerves were reconstructed. Except for the optic nerve and the missing olfactory nerve, all other cranial nerves demonstrated a normal anatomical course (Fig. 6). For comparison to normal anatomy, refer to Arnold and Kleiner [22]

### Cranial arteries

**Figure 7:**
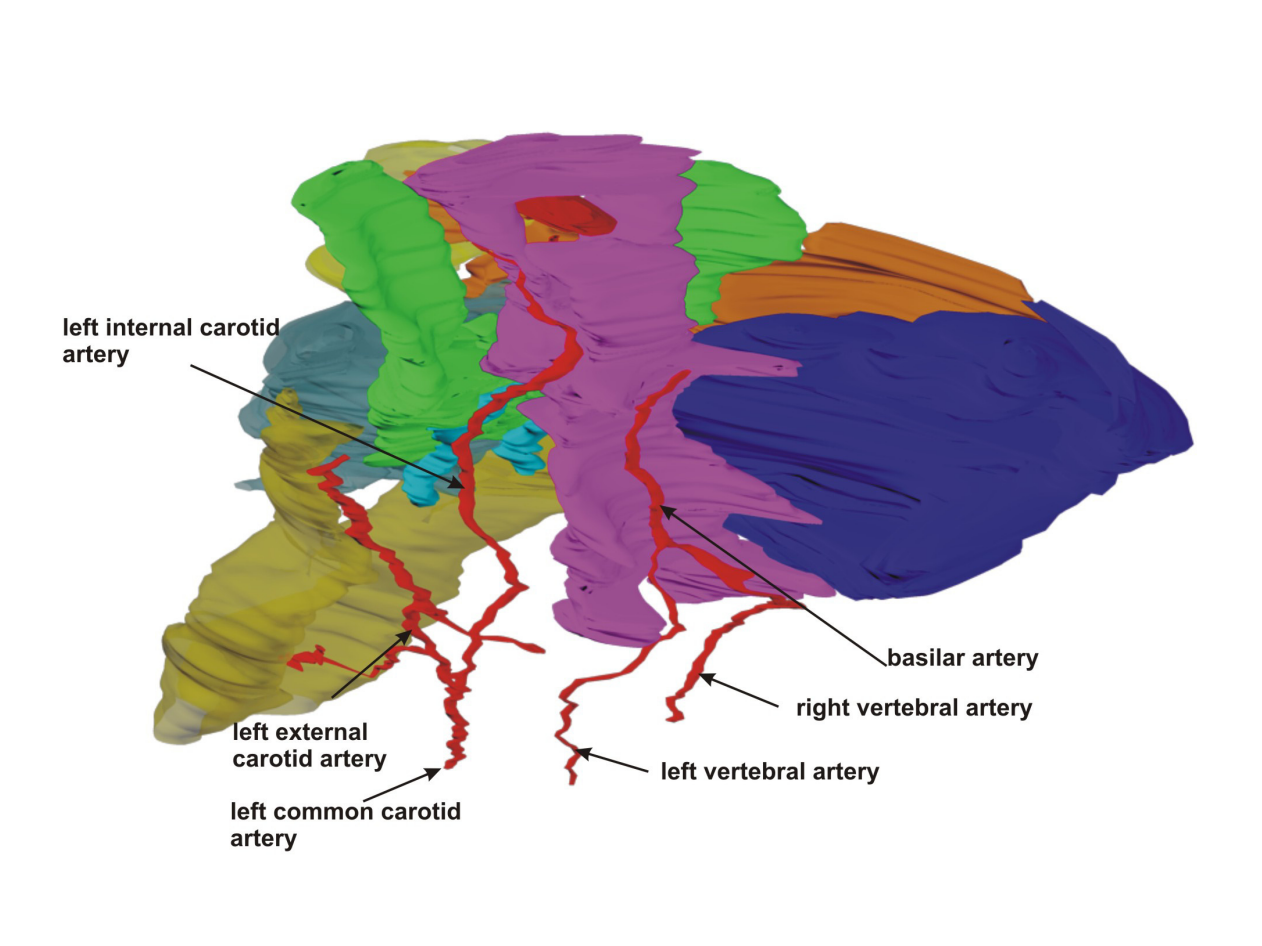
**Overview of main cranial arteries**. Left oblique view of a rendered 3-D reconstruction of cranial arteries. The temporal and zygomatic bones have been removed.

The external carotid artery showed a normal course and branching. The internal carotid artery could not be followed up until it met the basilar artery. The arterial circle of Willis could not be detected. The basilar artery could be reconstructed as far as the mesencephalon. In the middle of the clivus the basilar artery disappeared (Fig. 7). For comparison to normal anatomy, refer to Arnold and Kleiner [22].

### Orbit

**Figure 8:**
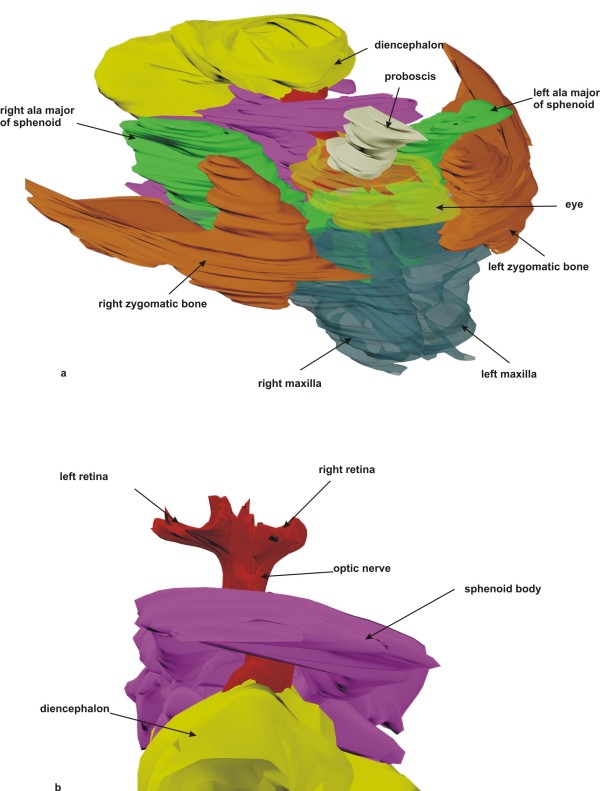
**3D reconstruction of orbital bones**. a) Right oblique upper view of the orbital bones containing a single eye and two retinae. The single optic nerve is connected with the diencephalon. b) Detail of the optic nerve with two retinae. The optic nerve is connected with the diencephalon leaving the orbit through one central optic canal.

The lateral borders of the single orbit were represented by the left and right zygomatic bones, and the bottom were the maxillary bones. The backside of the orbit was represented by the anterior side of the sphenoid body and laterally by both alae majores of the sphenoid. No bony roof of the orbit was present. The orbit contained a single cyclopic eye with two lenses and retinae (Fig. 8). From the two retinae a pair of optic nerves derived, which formed a single optic nerve behind the eye. This single optic nerve passed a single central optic canal and ended within the diencephalon. The optic nerve contained an inner cavity which continued into the diencephalon.

### Proboscis

**Figure 9:**
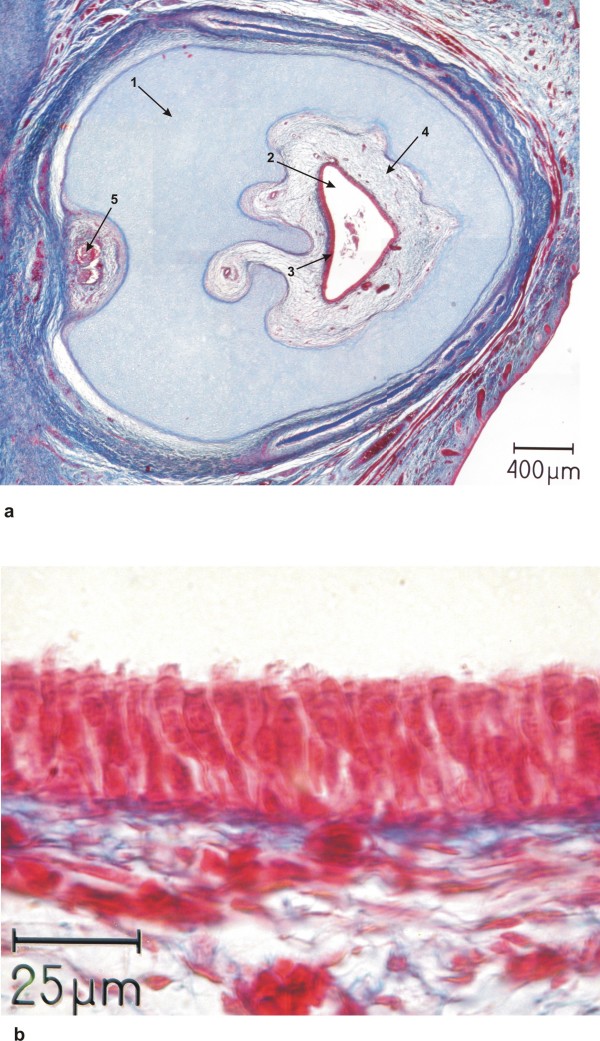
**Histology of proboscis**. a) Overview of a histological section of the proboscis showing the closed circular cartilaginous wall (arrow 1) with a central canal (arrow 2) with an epithelial lining (arrow 3) supported by loose connective tissue (arrow 4) which contains blood vessels and two nerves at the posterior side (arrow 5). b) Higher magnification of the epithelial lining of the central canal, depicting a respiratory epithelium with cilia (arrow 1) and goblet cells (arrow 2).

Above the cyclopic eye originated a single midline proboscis. The proboscis was represented by a circular wall of hyaline cartilage with a central canal. The inner lining of this central canal contained respiratory epithelium which is supported by loose connective tissue. Along the bottom of the proboscis a pair of nerves was found, but they did not enter the central canal (Fig. 9).

## Discussion

The gross anatomical features of the described case are similar to those which have been reported previously in the literature, such as a synophthalmic eye, an unpaired orbit, a proboscis above the eye and an alobar telencephalon[3-6,8,9,23]. This description of another holoprosencephalic case adds more information to the scarce literature about embryonic holoprosecephaly. One of the main features in the described case is the absence of a mesethmoid which, during normal embryogenesis, gives rise to the nasal capsule. The ethmoid complex is a derivative of the fronto-nasal process which develops from the prechordal mesoderm and divides the developing face into a left and right side. The ethmoid complex plays an important role in the lateralization of the paired structures of the face [24,25]. Failure in the development of the ethmoid has extensive consequences in facial development and results in malformations of the entire middle and upper face. A remnant of the missing ethmoid may be the proboscis above the eye. This structure is represented by a tube-like cartilage with a central canal which is lined by respiratory epithelium. Therefore, the term "nose-like," which has been used in the literature, [6,14,21] may be somewhat correct. If there is no definition of the median cranial plane, lateralization is impossible, and thus results in the expression of the holoprosencephalic phenotype. Thus holoprosencephly may be regarded as a failure in lateralization of the most anterior parts of the neural tube and the developing facial cranium.

The chorda dorsalis determines the bilateral symmetry and the pattern of the segmentation of the entire post-cranial body by the expression of Hox genes [26]. The skeletal derivatives of the cranial neural crest anterior to the chorda dorsalis are patterned through a combination of intrinsic differences between neural crest cells and extrinsic signals from adjacent tissues [27], and therefore are different from the rest of the body. The determination of the cranial median plane in early embryonic development may be under the control of various factors such as different genes, the most important of which is SHH [15,23,28,29], but environmental influences have also been implicated [28].

The incomplete arterial circle of Willis in this case is in accordance with other findings in holoprosencephly. It has been described that the arterial circle of Willis is incomplete in severe cases of holoprosecephaly [2,30] and may be related to the malformation of the central nervous system. The internal carotid artery disappeared just before it could enter the remnants of the telencephalon. The basilar artery could only be followed up to the anlage of the mesencephalon. The reason for the incomplete arterial supply may be, as in other cases, the malformation of the brain anlage.

## Conclusion

Holoprosencephaly seems to be a malformation of the most rostral parts of the brain and the cranium, as it affects mainly the telencephalon and the upper portion of the facial cranium. From the results of this investigation, and in combination with previous reports, it can be concluded that there is a defect in the definition of the median plane in embryonic development.

The histological finding of the proboscis with a respiratory epithelial lining leads to the conclusion that it is a nose-like structure and part of the fronto-nasal process which failed to develop properly.

It further can be concluded that the ethmoid plays an important role in the development of the bilateral symmetry of the face, and may play an important role in the determination of the median plane.

## Competing interests

The authors have no competing interests as the research has been carried out with University funds.

## Authors' contributions

WHA: Identified the histological structures, supervised the project and wrote the manuscript.

VM: Did the 3-D reconstruction.

Both authors read and approved the final version of the manuscript.
